# The Central London Throat and Ear Hospital

**Published:** 1892-10-22

**Authors:** Richard Kershaw


### THE CENTRAL LONDON THROAT AND EAR HOSPITAL.

Sir,—In your issue of October 1st you have been good enough to refer to two letters, one by Mr. Lennox Browne and one by myself, to which I would ask to be allowed to send a short rejoinder. In the first place, I am glad to learn that although in your foot-note to my letter of August 6th, you stated that "the estimated expense of the out-patients, at this hospital, is excessive, when compared with the slight outlay of the department," you did not intend to convey any charge of extravagance against our work. It seemed to us the fairest way to take the average cost of the fifty-five special hospitals, which, amounting to 3s. 10¾d., was higher than that of the Central Throat and Ear Hospital, which was 3s. 6½d.; but since you consider "the only fair way is to classify the various special hospitals into groups, and to compare the expenditure of these in each group," I would draw your attention to the cost of the one other throat hospital and the four chest hospitals, the work of which is the nearest approaching to my own. The following are the figures: 3s. 2d., 2s. 11d., 3s. 1½d., 4s., 4s. 8½d.—an average of 3s. 7d. With regard to the other throat hospital, I would point out that while the cost of their out-patients is returned at 4d. per head less than ours, their in-patients show an excess of 4s. per head per week, while at three out of the four chest hospitals the cost is also very much greater than with us. I would, in conclusion, again refer to the economy exercised in our administration, which, I think, is deserving of as much praise as that lavished upon other institutions.—I am, your obedient servant, Richard Kershaw, Secretary.—October 12th, 1892.

